# "Cervical Cancer—A Silent Disease in the Community”—A Qualitative Study on Awareness of Cervical Cancer in Tanzania

**DOI:** 10.1177/23779608251393079

**Published:** 2025-11-07

**Authors:** Chucki Christina Mtuya, Jenny Cadstedt, Janet Mattsson, Hélio Adelino Manhica, Furaha Serventi, Rogathe Machange, Paulo Kidayi, Declare Mushi, Gunilla Björling

**Affiliations:** 1 School of Nursing, 108094 KCMC University, Moshi, Tanzania; 2 Department of Health Sciences, The Swedish Red Cross University, Stockholm, Sweden; 3 Department of Neurobiology, Care Sciences, and Society, 620467 Karolinska Institutet, Stockholm, Sweden; 4 Faculty of Health and Life Sciences, Linnaeus University, Växjö, Sweden; 5 Department of Health and Care Sciences, The University of Southeast Norway, Drammen, Norway; 6 Cancer Care Centre, 108095 Kilimanjaro Christian Medical Centre, Moshi, Tanzania; 7 School of Public Health, KCMC University, Moshi, Tanzania; 8 School of Health and Welfare, 4161 Jönköping University, Jönköping, Sweden

**Keywords:** cervical cancer, awareness, prevention, screening, HPV vaccination, low health literacy, health literacy

## Abstract

**Introduction:**

Cervical cancer (CC) remains a leading cause of morbidity and mortality among women in Sub-Saharan Africa, particularly in Tanzania, despite being preventable through screening and human papillomavirus (HPV) vaccination. Although national strategies exist, uptake remains low. This study explored awareness of CC screening, care, and vaccination among men and women in both urban and rural areas of Kilimanjaro, Tanzania.

**Method:**

A qualitative descriptive design was conducted from April to May 2024. Four Focus Group Discussions with a total of 31 participants (including men and women) were conducted in both urban and rural communities. Simple random sampling was used to select the participants. A semistructured guide covered CC awareness, vaccination, screening, and community engagement. Transcripts were translated, coded, and categorized. Inductive content analysis was used. The study report used Consolidated Criteria for Reporting Qualitative Research guidelines.

**Results:**

Participants showed limited knowledge of CC, its causes, and the benefits of HPV vaccination and screening. Three main categories with eight subcategories emerged: (1) Low health literacy, (2) Challenges in accessing CC prevention, and (3) Community involvement. Myths (e.g., vaccination causing infertility), healthcare system barriers, financial constraints, and stigma contributed to poor uptake. Male and opinion leader involvement was identified as crucial, but both groups lacked accurate information and were not actively promoting CC prevention.

**Conclusion:**

This study highlights limited knowledge and persistent misconceptions about CC and its prevention among men and women in both urban and rural areas of Tanzania. Structural and sociocultural barriers, including low health literacy, financial constraints, gender norms, and misinformation, hinder access to screening and HPV vaccination. Engaging male partners, opinion leaders, and communities through targeted education and improved health communication is essential. These findings provide foundational knowledge to inform policy and design context-sensitive interventions to reduce the CC burden in Tanzania and similar low-resource settings.

## Introduction

### Prevalence

Cervical cancer (CC) is a major global public health issue and the fourth most common cancer among women of reproductive age (Binka et al., 2017; Bray et al., 2018). Over 570,000 new cases and 311,000 deaths occur annually, projected to reach 700,000 cases by 2030 without stronger prevention (Arbyn et al., 2020; Barrow et al., 2020; Runge et al., 2019; WHO, 2022). Sub-Saharan Africa (SSA) bears the highest burden, with 20% of global cases and 24% of deaths (Griesel et al., 2021), and mortality could rise by 50% by 2040 without expanded vaccination and screening (Barrow et al., 2020). In Tanzania, CC is the leading cancer among women, with an incidence rate of 59.1 per 100,000, well above the global average of 13.1 (Bruni et al., 2019, 2022; Gesink et al., 2020). Late-stage diagnosis (Stage III or IV) contributes to poor outcomes, with nearly 7,000 deaths annually (Moshi et al., 2018; Runge et al., 2019).

### Prevention

Human papillomavirus (HPV) is the primary cause of CC and can be effectively prevented through screening and vaccination, potentially lowering incidence and mortality by up to 80% (Bénard et al., 2023). However, global vaccine coverage remains low, with only 27% of the target population receiving the first dose well below the 90% target set for 2030 (Simelela, 2021; WHO, 2022). A multicountry study across 78 low- and middle-income countries (LMICs) estimated that full implementation of the WHO 90-70-90 elimination strategy could reduce CC mortality by almost 99%, potentially saving more than 62 million women's lives (Canfell et al., 2020)

In LMICs, CC accounts for over 90% of related deaths (Arbyn et al., 2020; WHO, 2020), yet only 3% of eligible girls receive the HPV vaccine and 44% of women undergo screening (Duncan, 2022). The HPV vaccine uptake in Africa varies from 34% to 93.3%, and safe vaccines combined with screening could reduce incidence by 90% (Gavi, 2021); Gultekin et al., 2020). In East Africa, coverage is only 35%, underscoring the urgent need for improved implementation (Agimas et al., 2024). In Tanzania, CC prevention efforts include the National Cervical Cancer Prevention and Control Strategic Plan (United Republic of Tanzania, Ministry of Health and Social Welfare, 2015) and the introduction of the HPV vaccine program in 2018 (Mphuru et al., 2022). Nevertheless, uptake remains low (Nhumba & Sunguya, 2022), with national screening coverage at just 7.28% (Asgedom et al., 2024) and only 6% of women screened in the Kilimanjaro region (Cunningham et al., 2015). Low screening rates, especially among asymptomatic women, pose a major challenge to achieving the WHO 90-70-90 elimination targets by 2030 (Gultekin et al., 2020; Stefan et al., 2022;WHO, 2022).

### Review of Literature

Prevention, early treatment, and raising awareness of CC are crucial to reducing the disease burden. Low levels of CC screening and HPV vaccination are linked to poorer awareness of available prevention services in rural compared to urban areas. Contributing factors include the absence of screening and HPV vaccination services in rural settings, low health literacy on CC and HPV infection/vaccination (Zhetpisbayeva et al., 2023), low coverage of CC screening and vaccination, financial constraints from out-of-pocket expenses, and geographical barriers (Appiah et al., 2025), all limiting access to healthcare services in rural areas. Social and cultural barriers (Asgedom et al., 2024) are also more prominent, while a shortage of qualified practitioners further reduces participation in prevention strategies in rural settings (Appiah et al., 2025; Banks et al., 2022).

Patel et al. (2020) found that low awareness among women led to poor screening uptake. In SSA, awareness of CC is generally low, especially in rural areas where 60–80% of cases occur (Drokow et al., 2020; Moshi et al., 2018; Osei et al., 2021). A lack of knowledge about prevention increases CC risk (Binka et al., 2017). Many women are unaware of CC symptoms, causes, or screening options before diagnosis, with some patients reporting shock upon learning about HPV (Demissie et al., 2022; Jibat et al., 2024). Low public awareness exacerbates CC morbidity and mortality by hindering the uptake of preventive measures (Binka et al., 2017; Shrestha et al., 2020). Education campaigns have shown success in correcting misconceptions, increasing screening, and improving awareness about CC and HPV vaccination (Cooper et al., 2021; Osei et al., 2021). In Tanzania, myths about HPV affecting fertility, spread by community leaders, have further limited vaccine uptake (Cinya & Mubangizi, 2024). A study in Kilimanjaro found that although many women were aware of CC, only 6% had been screened, and knowledge of HPV vaccination was low (Cunningham et al., 2015). There are limited qualitative research on community-level factors influencing CC awareness, as most studies are facility-based in SSA. Therefore, this study aimed to explore awareness of CC screening, care, and vaccination in urban and rural areas among men and women in Tanzania.

## Method

### Study Design

This is a qualitative descriptive design, employing inductive content analysis to investigate awareness of CC screening, care, and vaccination among men and women in urban and rural areas of Tanzania. Data were collected through Focus Group Discussions (FGD; Krueger & Casey, 2015), a method suitable for capturing in-depth public insights. This approach was considered appropriate as qualitative methods are particularly effective for understanding participants’ experiences and perspectives of CC (Creswell & Poth, 2016). The study adheres to the Consolidated Criteria for Reporting Qualitative Research checklist, focusing on aspects such as research team reflexivity, study design, data analysis, and findings (Tong et al., 2007), see Appendix 1.

### Research Questions

- Are women and men in urban and rural areas of Kilimanjaro aware of CC screening, care, and vaccination?
- What are the contextual and sociocultural factors that influence awareness and uptake of CC prevention services in these communities?

### Sample

A simple random sampling approach was used to select the study setting and participants. The study participants were men and women aged 18 and above in the Kilimanjaro region, which consists of seven districts. Two districts, Moshi Urban and Moshi Rural, were purposively chosen due to their high CC prevalence. The sample was drawn from communities with a high prevalence of CC, specifically the Moshi Urban and Moshi Rural districts of the Kilimanjaro Region, selected to capture populations most affected by the disease. According to data from the Kilimanjaro Population-Based Cancer Registry, the overall prevalence of CC in the region is 11.6%, with Moshi Rural exhibiting the highest prevalence at 57% (Kilimanjaro Christian Medical Centre, Cancer Care Centre, 2022).

#### Recruitment of Participants for FGD

From each district, two wards were randomly selected in Moshi Urban and Moshi Rural. In each ward, the respective Ward Executive Officer was contacted by telephone and appointments were arranged to present the study's purpose. Thereafter, one street in the urban area and one hamlet in the rural area were randomly chosen. The ward executive officer informed the local street chairpersons about the study and they, in turn, invited 10-cell leaders (local leaders) and introduced the principal investigator (PI). Using household lists provided by these leaders, eligible households were identified through systematic sampling. From each household, one adult (man or woman aged 18+) was randomly selected from a list of adults in the home. This process ensured a diverse and representative community sample. Selected participants were contacted by phone and invited to participate in FGDs the following day.

A total of 31 participants were recruited for the FGDs. Four face-to-face FGDs were conducted: two groups consisting of male participants and two groups consisting of female participants in urban and rural areas, respectively. Each group included members from different age brackets, ranging from 18 to 69 years, and comprised between 5 and 11 individuals. This diverse composition allowed for a broad representation of perspectives across genders and age groups (Krueger & Casey, 2015). All participants provided written informed consent and participation was voluntary. All participants were eligible to participate in the study regardless of their CC status. However, none of the participants had CC.

#### Inclusion Criteria

Men and women >18 years of age (without considering their CC status) and those who could speak Swahili.

#### Exclusion Criteria

Those who were not around during the data collection period were excluded from the study and those who did not provide informed consent.

### Ethical Considerations

The study adhered to the guidelines on research on human subjects. The Declaration of Helsinki (WMA, 2013) ethical clearance certificates were obtained from the KCMC University Research Ethics Review Committee and the National Institute for Medical Research, Tanzania. The directors of the respective study sites granted permission. Written informed consent was obtained from participants, and participation in the study was voluntary. All information collected was handled at a high level of confidentiality. The participants had the freedom to withdraw anytime during the study without any negative consequences.

### Data Collection

A semistructured FGD guide, (Appendix 2), was developed by researchers based on the study's objectives (Krueger & Casey, 2015). It included sections on sociodemographic characteristics, general awareness of cancer and CC prevention, risk factors, signs, and symptoms. The interview guide was initially created in English and translated into Kiswahili by bilingual researchers, followed by back-translation to ensure linguistic suitability for the community (Behr, 2017). The tool was piloted among male and female community groups of seven participants to assess the understanding of the guide. Only minor adjustments, such as typographical corrections and resequencing of questions, were made.

Data collection took place between April and May 2024. The same interview guide was used for all participants in every session. All FGDs were conducted face-to-face in the respective ward office conference room, with participants seated in a semicircle arrangement. All participants signed informed consent forms before the discussions started, following Marshall and Rossman (2016).

To maintain confidentiality, each participant was assigned a number displayed on paper for identification purposes. With participant consent, the FGDs were recorded using two digital recorders to ensure audio backup. The FGDs were moderated by two facilitators: for female groups, the first author (PhD student with an MSc in public health) and a female moderator; for male groups, a male social scientist with a master's degree in public health and extensive experience in qualitative research.

In the female FGDs, the social scientist acted as the note-taker, while the first author took notes during the male FGDs. All facilitators were assistant lecturers at the time of data collection. Each discussion lasted between 60 and 90 min. Data were collected only once, and transcripts were not returned to participants for review, though member checks were employed to enhance data accuracy. After each FGD, transcription and translation were completed, and data analysis began immediately, including data familiarization. Data saturation was reached after the third FGD in rural areas; however, the researchers agreed to conduct an additional (fourth) FGD in urban areas to confirm whether any new information would emerge.

### Data Analysis

Data was analyzed using content analysis with an inductive approach inspired by Graneheim and Lundman (2004). The inductive content analysis was chosen, as the field was unexplored. This approach was chosen to explore the phenomenon directly from the participants, rather than being predetermined (Polit & Beck, 2017). The FGD audios were transcribed verbatim in Kiswahili. The transcripts were validated, meaning checked for quality, rigor, and relevance as per the audios, and later translated from Kiswahili into English by a bilingual Tanzanian for easy joint analysis for the whole research team and those who do not understand Kiswahili. The researchers read the transcripts several times to understand and be conversant with the transcripts to ensure the data quality. Checking the Kiswahili and English have the same meaning was also done before going to the next step of coding and analysis. The next step started with marking meaningful units in the transcripts. These were then condensed and given codes. Data was coded by three independent researchers. The codes containing similar meanings were developed into eight subcategories. Through this inductive analysis, developed by Graneheim and Lundman (2004), the subcategories were merged into three main categories. The researchers reached a consensus in the last step, see Table 1.

**Table 1.** Composed Main Categories, Subcategories, and Codes.

	Main category	Subcategory	Codes
1	Low health literacy	*The silent disease*	No one is speaking in the community about CC and CCS
			A community not aware is at risk of CC
			Lack of information on CC and HPV vaccine
			Discuss other diseases such as HIV, UTI, and fungus, but not CC and CCS
		*Myths and traditional beliefs about CC*	believes HPV vaccination will cause infertility later
			Cervical cancer is not preventable; only possible through prayer
			CC inherited from the family, out of is no risk of the disease
			Healthcare provider touches the cervix during CC screening
			Touching the cervix during screening increases the risk for CC.
			The uterus is pulled out, and then will be pushed in after CC screening
			CC induces pain in the uterus, & it is embarrassing!
2	Challenges in accessing cervical cancer prevention	*Healthcare facilities barriers*	Long waiting time at the health facility,
			the distance to reach the facility
			Wasted time due to the long walk to the facility Time spent accessing urban services
			The screening procedure disappoints women from going for screening
		*Family and community barriers*	Women don't have time for CC screening
			Masculinity hinders women's decision-making for CCS
			Travel cost for fare, even though service will be free/cost implications
			Not feeling sick, no need to go for CC screening
			a habit of not attending the hospital for any service if not sick
			Women are busy earning money for a living/bread breadwinners/economic reason
			Women are worried about what they will eat if they go for screening
		*Fear of abandonment*	A man runs away if the CCS tests positive
			A woman can go for screening and still fear disclosing in the community
3	Community involvement	*Male involvement*	The cultural practice
			Influence on decision making
			Couple involvement for family CCS and vaccination
		*Involvement of opinion leaders*	Government leaders trust
			Religious leader trust
			Influential people

CC: cervical cancer.

### Rigor

To establish trustworthiness and rigor of the findings and research free from researcher bias, study's adherence to the trustworthiness criteria: credibility, dependability, transferability, and confirmability (Stenfors et al., 2020). Credibility was achieved by letting the data guide the analysis, supported by diverse samples from rural and urban areas, enhancing representativeness. Dependability, or data stability over time and conditions (Elo et al., 2014), was ensured through rigorous methods, including independent analyses, transcript validation, and bilingual translations. Inductive content analysis, participant quotations, and consensus on themes ensured confirmability. Transferability was strengthened by involving participants from varied settings, making the findings applicable to similar groups and contexts (Lincoln & Guba, 1985).

## Results

### Sociodemographic Results

A total of 31 participants (16 females and 15 males) took part in four FGDs. Of these, 20 participants were from urban settings and 11 from rural areas, ensuring a diverse representation of perspectives across gender and geographical contexts. Further details of the participants’ characteristics are provided in Table 2.

**Table 2.** Socio Demographic Characteristics of Participants n=31

**Variable**	**Frequency**	**Percentage**	**Mean± SD**
Age			45±13
Age groups			
*23-40*	12	38.7	
*41-50*	10	32.3	
*51-69*	9	29.0	
Total		100.0	
Gender			
*Male*	15	48.4	
*Female*	16	51.6	
Total		100.0	
Marital Status			
*Married*	23	74.2	
*Unmarried*	8	25.8	
Total		100.0	
Tribe			
*Chagga*	10	32.3	
*Pare*	10	32.3	
*Sambaa*	7	22.6	
*Other*	4	12.9	
Total		100.0	
Residence			
*Rural*	11	35.5	
*Urban*	20	64.5	
Total		100.0	

### Research Questions Results

Regarding the research questions, the content analysis generated 20 codes from the raw data, eight (8) subcategories, and three (3) main categories: *Low health literacy*, *Challenges in accessing CC prevention*, and *Community involvement*. The three (3) main categories with eight (8) subcategories, as well as the codes, are presented in Table 1.

### Low Health Literacy

This category highlights the generally low level of awareness regarding CC among participants across all groups. Many individuals were unaware of the causes of the disease and had never heard of HPV, HPV vaccination, or associated risk factors. The subcategories within this category are: *The silent disease* and *Myths and traditional beliefs about CC*.

#### The Silent Disease

Most participants reported that discussions about CC are virtually absent within the community. Although there is a general sense of concern regarding the threat posed by CC, the community lacks detailed knowledge about the disease. As a result, individuals are unsure of what to discuss and remain silent, lacking the confidence to address the topic due to insufficient awareness. Male participants, in particular, noted that conversations about CC are rare because people generally do not know what information to share with friends or neighbors during social interactions. This silence was, however, observed among both women and men, in both rural and urban areas, rendering CC a “silent disease” within the community and underscoring the low level of awareness, as illustrated in the excerpt below.

Women are not aware of CC, because when you sit with the majority of the community surrounding us, especially the middle aged, you cannot hear them talking about CC that is caused in this way and that way. The government is silent, the community is silent, and the family is silent. The leaders in the community don't know, so they don't talk to the community, and everyone in the community is quiet! (FGD 2; Male rural**)**

#### Myths and Traditional Beliefs About CC

All groups revealed a lack of coherent understanding of HPV vaccination, largely due to the spreading of incorrect information about CC prevention within the community. Participants expressed concerns and uncertainty regarding the government's recommended strategies for CC prevention, including vaccination and screening, as a result of ambiguous narratives and rumors circulating within the community. One widespread rumor suggested that the vaccines had been imported from abroad with the intent of causing infertility, allegedly targeting young girls to prevent them from having children. This misinformation led many parents and guardians to reject the vaccination of their daughters, often keeping them at home on vaccination days to avoid participation in the program.

But for sure, many parents from our street said - don't vaccinate your children. They will not be able to bear children at all if vaccinated. We agreed in the community that we should stop children from going to school on that day because they will not bear children. After all, HPV vaccination kills all the eggs. (FGD4; Female urban)

Furthermore, participants in the male groups expressed the belief that CC is hereditary and cannot affect an individual unless there is a family history of the disease. They explained further that if they trace their family history and find no evidence of CC among relatives, they feel unconcerned about the risk. Consequently, this perception leads participants to believe that vaccination and screening are unnecessary for preventing the disease.

Others believe it is something that is inherited from the family, so it is not for them. They think they can't get the disease because of no history of the disease in their family. So, they take it easy; they don't take it seriously. (FGD1; Rural male)

Participants also shared several myths about the CC screening procedure, influenced by rumors circulating within the community. One widely held belief was that during screening, the uterus is temporarily removed to obtain a sample and then returned to the body. Additionally, women's groups in both rural and urban areas perceived the screening process as dangerous, based on the belief that taking a biopsy leaves a scar on the cervix, potentially causing harm.

I heard that when you go for screening, the uterus is pulled out and then will be pushed in after screening. Yes! I heard women gossiping at the veranda lots of stories one saying, yes, when you go for screening the uterus pulled out and put hanging there and picked and picked! You see my mother died! You see like that, if am very fine, will I go? (FGD 1; Female rural)

Concerning prevention and treatment for CC, participants expressed the belief that the disease cannot be prevented or cured. They stated that available medical interventions are solely for palliative care, aimed at alleviating symptoms rather than providing a cure. Furthermore, the community widely held the view that prayer is the only means of preventing CC.

Altogether, there is no treatment for CC, you will go and be given medication to help you add some days to life. Yes, I repeat, no medication. The only medicine there is to help you …. is life-prolonging, but for cancer, in general, there are no treatments. We need ONLY to pray to our God to help us! (FGD2; Male rural)

### Challenges in Accessing CC Prevention

Several barriers were identified as hindering the utilization of CC prevention services and efforts within the community. Personal, family, healthcare-related, and community-level barriers were highlighted as important challenges in accessing CC prevention. For instance, many women reported a lack of time due to busy schedules spent earning a livelihood and fulfilling household responsibilities. Additionally, cultural barriers were noted, as some women required their husbands’ approval to access prevention services. However, many husbands were unaware of the importance of CC prevention, further complicating efforts to address the issue. These challenges are evident in the three subcategories: *Inadequate healthcare facilities*, *Family and community barriers*, and *Women's health versus financial priorities*.

#### Healthcare Facilities Barriers

In the women's FGDs, participants narrated that when they visit healthcare facilities and hospitals, healthcare providers often attribute health concerns solely to urinary tract infections (UTIs) or intestinal worms, which are perceived as the most common health issues in the community. This limited diagnostic approach means that further investigation into other potential conditions, such as CC, is rarely pursued. Moreover, CC screening services are unavailable at local healthcare centers, with UTI and deworming treatments being the primary services readily accessible to the community. Participants also highlighted that accessing CC screening services requires traveling to distant healthcare facilities, which involves additional costs, such as travel expenses, creating a significant barrier. Furthermore, the time needed to walk long distances to these facilities contributes to delays in seeking CC-related healthcare services, as women often feel that the effort and time investment are burdensome leading to a feeling of wasted time.

UTI, or worms is what most healthcare providers think is the problem and is what they treat before doing a thorough assessment! Yes, no further diagnosis because the provider will be treating UTI but you might have been affected with CC already you don't know it grows. You find the next day you go with the same complaint, and you are given the same diagnosis and medication, and the problem becomes big! (FGD 1; Female rural)

#### Family and Community Barriers

The current situation, characterized by a lack of parental involvement in school-based vaccinations for CC, results in parents preventing their children from receiving the HPV vaccine. This issue was particularly noted during the FGDs with women in rural areas.

… You know this thing about vaccination comes from school through our children, but we don't know anything as parents, as we are not informed and not involved as parents. This causes some parents to prevent their daughters from going to school during the day of vaccination. You find the vaccination might be very important, but a parent prevents a child from getting vaccination because we don't know the alpha or omega. (FGD1; Female rural)

The lack of effective health education on CC within communities was identified by participants as a barrier to CC prevention initiatives. Participants explained that without adequate information about screening and HPV-vaccination, community acceptance and uptake of these preventive measures are likely to remain very low.

Lack of information/awareness on HPV vaccination is a barrier … but if people get proper information and awareness … no one will hinder acceptance of the vaccine, … if they get an education they will go for screening … if not, they won't go, and even currently, they are not going because they are not aware. (FGD1; Female rural)

#### Women's Health Versus Financial Priority

The participants narrated that they are busy earning some money for their daily living and do not have time for health checkups. For women, not yet sick, health is not an immediate priority as financial priorities come first, to get money for daily food.

… I don't have time - instead of going for screening, … I'd better go to the farm and look after my rice paddy and my tomatoes, … I want to go to “Manyema” (a local market in Moshi), and earn a living. The time I go for screening, what will my children and I eat? That is what causes women from Kilimanjaro not to go for testing/screening. (FGD4; Female urban)

Furthermore, it is not common practice in the community to go for health checkups without being sick. The custom is that people wait until they feel sick; to go to the hospital for treatment remedy is the normal practice among the participants. Furthermore, it happens that if somebody plans to go for screening or to vaccinate the daughter, the community discourages it because going to the hospital is common only for those showing sickness but not for those appearing healthy.

The problem for us people in the lower class, we wait until a person gets sick and is told having this disease. But you cannot see a person going only for check-ups, for screening without being sick (laughing)! Screening is money and she doesn't have money, most in the community wait until they get sick is the time they go for treatment. (FGD2; Male rural)

#### Fear of Abandonment

The female participants stated fear of abandonment as a reason which hinders the utilization of CC preventive services. If they would receive a positive result after screening, they fear that they will be abandoned by their husbands. To be diagnosed with CC is seen as a stigma in the community, which leads to abandonment.

Also, women are afraid of testing/screening. I saw one woman who went for screening, and she told her husband that I went for screening and was found to have CC symptoms. They continued to live, and the husband had to run away from that woman. “As she has that disease, she is of no use to me,” he runs away. So, a woman can go for screening and still fear to disclose in the community. They are afraid because many marriages have been broken because of CC (FGD4; Female urban).

### The Role of Community Involvement

Community involvement plays a major role in any program intervention. Participants narrated there was a low level of involvement, and most of the stakeholders were not involved and informed about CC screening and vaccination. Within this category, this is further developed in the subcategories of *male involvement* and *involvement of community leaders*.

#### Male Involvement

The participants narrated that male involvement in CC prevention could significantly enhance support for CC screening and vaccination. Culturally, men are regarded as heads of the family and primary decision-makers, giving them substantial influence over their wives’ and daughters’ acceptance of CC prevention measures. Male participants explained that women cannot independently decide to undergo screening or take their daughters for vaccination without their husbands’ approval.

Men expressed that women could not decide to go for screening or even take their young girls for vaccination without their husbands’ approval, and educating men on CC prevention will enhance women’s habit of going for screening and vaccination. They further emphasized that educating men about CC prevention could encourage women to adopt regular screening habits and ensure their daughters receive the HPV vaccination without their husbands’ approval.

Involving both males and females in awareness about the prevention of cervical cancer will influence women/wives to go for screening and vaccination to prevent CC because men have power in decision-making. He is the head of the family, and women cannot just decide to go for screening for CC. They will only go if their husbands sensitize them to go. (FGD2; Male rural)

The quote above shows that women are vulnerable and dependent on their husbands’ understanding of the severity of CC and their approval to seek healthcare**.**

#### Involvement of Opinion Leaders

Participants emphasized the importance of involving local government and religious leaders in promoting acceptance of HPV vaccination and screening within the community, as these leaders are often more trusted to deliver culturally sensitive information than healthcare providers. However, it was noted that local leaders rarely address the topic of CC due to a lack of adequate information. Engaging these leaders in disseminating accurate information about CC could considerably enhance community acceptance of prevention strategies, as they are highly trusted as representatives of the government.

If the government leader passes over the announcement to insist on CC screening and HPV vaccination, this will reassure the community, because the leader could not accept something that destroys the community. You will go and tell your child to go for that vaccination! Yeah, because you have proper information from your leader in that community! (FGD2; Male-rural)

They can inform us through the village leaders, and we will understand vaccination. (FGD 1; Female rural)

## Discussion

This study investigated awareness of CC screening and vaccination among men and women in urban and rural areas of the Kilimanjaro region, Tanzania. Findings revealed three main categories: Low health literacy, Challenges in accessing CC prevention, and Community involvement. There is a critical need to address health literacy to dispel prevalent myths about screening and vaccination. Barriers such as healthcare infrastructure limitations, family dynamics, and community factors significantly impede service utilization. Engaging men and community opinion leaders could promote greater acceptance of vaccination and screening programs.

### Low Health Literacy

The study found low health literacy on cervical CC, with limited knowledge and confidence to discuss the disease. Poor public awareness underscores the need for better information dissemination, such as brochures and social media, which have proven effective for CC prevention (Baharum et al., 2020; Petersen et al., 2022). Unlike prior studies, participants were largely unaware of risk factors, including HPV and its vaccine (Chelva et al., 2024; Demissie et al., 2022; Drokow et al., 2020; Jibat et al., 2024; Lin et al., 2020; Rick et al., 2019), though women in both urban and rural areas identified multiple childbirths as a risk factor, an understanding absent among men. In contrast, Swai et al. (2023) found high awareness of CC risk factors, HPV, testing eligibility, and screening benefits, likely due to their hospital-based setting. Okyere et al. highlight the need for community sensitization and behavioral change to improve vaccination and screening uptake. Extensive campaigns have enhanced public confidence in discussing health issues, and similar efforts could boost CC awareness, increase preventive service use, and reduce CC-related morbidity and mortality, contributing to economic growth.

Participants in the present study believed that HPV vaccination causes infertility among their daughters, discouraging its acceptance in both urban and rural communities. This misconception, prevalent among women's groups, reflects a lack of awareness regarding the vaccine's role in preventing CC, despite the WHO (2024) confirming its safety and efficacy. Similar myths associating the vaccine with infertility or family planning have been reported in other studies (Chona et al., 2023; Hasahya et al., 2016). Additional concerns about vaccine complications, as noted by Almehmadi et al. (2019) and Drokow et al. (2020), further contribute to rejection. Dispelling these myths and emphasizing the vaccine's benefits is essential for increasing uptake and reducing the CC burden.

Misinformation about CC screening was found in this study, which limits women's access to preventive services. False beliefs increase fear and reduce screening uptake in rural and urban areas (Chona et al., 2023; Cunningham et al., 2015; Petersen et al., 2022). Some participants incorrectly believed that the cervix pulled out during screening, which reflects a lack of accurate information, also confirmed in research (Hasahya et al., 2016). Furthermore, the perception of CC as solely hereditary lowers perceived risk, particularly among men in rural areas, who see no need for vaccination or screening. This aligns with findings by Adedimeji et al. (2021). Addressing these myths through community education could help meet WHO's (2020) goal of screening 70% of women aged 35–45, enabling early CC detection and treatment in LMICs.

### Challenges in Accessing CC Prevention

This study identified key barriers to CC prevention, including inadequate healthcare infrastructure, limited parental involvement in HPV vaccination, financial constraints, cultural factors, and fear of abandonment. Rural women faced added challenges such as long travel distances, transport costs, and time spent accessing urban screening services, consistent with earlier studies (Asgedom et al., 2024; CDC, 2024; Gizaw et al., 2022; Hussein et al., 2024; Isaacson et al., 2023; Srinath et al., 2023). Long wait times and associated costs also deterred use, particularly in rural areas where facilities are less equipped (Adedimeji et al., 2021; Bensemmane et al., 2022; Chapola et al., 2021).

Parental involvement was a barrier, especially among rural women and urban men, with parents feeling excluded from HPV vaccination decisions for their daughters, leading to hesitancy (Ho et al., 2022; Mihretie et al., 2022; Osei et al., 2021; (Tobaiqy & MacLure, 2024) For rural women, cultural beliefs and low education may discourage provider engagement, while urban men's family health concerns drive their interest in inclusion. In contrast, higher parental acceptance has been reported when providers offer information (Anyaka et al., 2024; Davies et al., 2021). Strengthening parent–teacher partnerships and public awareness could improve uptake.

Financial difficulties significantly impacted healthcare prioritization, with urban men and women reporting that daily expenses took precedence over health, consistent with findings by the Osei et al. (2021), and Adler et al. (2023). Cultural norms favoring health-seeking only when symptomatic further lowered screening and vaccination rates (Adler et al., 2023; Tapera et al., 2019).

Fear of abandonment was another barrier, especially among urban women, who feared stigma and partner rejection if diagnosed with HPV or CC. This fear, often linked to low health literacy, reflects findings from other studies where women faced blame or abandonment after diagnosis (Chona et al., 2023; Dirar et al., 2022; Morse et al., 2023). Such concerns discourage screening and treatment, perpetuating low uptake (Bateman et al., 2019; Defo & Domgue, 2020; Ragan et al., 2018; Srinath et al., 2023).

### Community Involvement

Engaging men in health promotion is crucial for effective CC prevention. This study found that rural and urban participants supported male involvement in HPV screening and vaccination to boost acceptance and uptake (Tobaiqy et al., 2023). Men were willing to support partners and vaccinate daughters if provided accurate information, consistent with findings from Uganda (de Fouw et al., 2023). Women's participation in CC screening is often shaped by male partners, who control resources and grant service permission, particularly in African contexts where men are key decision-makers (Adedimeji et al., 2021; Chapola et al., 2021; Chelva et al., 2024; de Fouw et al., 2023; Drokow et al., 2020; Jibat et al., 2024; Mantula & Toefy, 2023).

Opposition from male partners can hinder uptake, with married women sometimes less likely to be screened (Adewumi et al., 2019; Gizaw et al., 2022; Srinath et al., 2023), highlighting the need for effective male engagement. Involving trusted community leaders, such as religious and government figures, can also improve acceptance of CC prevention (Adewumi et al., 2019; Mantula & Toefy, 2023; Tapera et al., 2021). However, this study found these leaders were not actively promoting such services, likely due to low health literacy and misconceptions that can hinder HPV vaccine uptake (Cinya & Mubangizi, 2024). Training and sensitizing opinion leaders is therefore critical for improving outreach, acceptance, and early detection.

### Study Strengths and Limitations

This study provides valuable insights into community experiences with CC prevention by incorporating perspectives from both men and women. Two skilled qualitative researchers (male and female) conducted focus groups, ensuring participant views were well represented (Creswell & Poth, 2016). Adherence to trustworthiness criteria, credibility, dependability, transferability, and confirmability (Stenfors et al., 2020) was achieved through diverse sampling, rigorous procedures such as transcript validation, bilingual translation, and independent coding (Elo et al., 2014), and consensus-based inductive analysis (Lincoln & Guba, 1985). While limited to specific geographic areas and subject to the inherent subjectivity of qualitative research (Polit & Beck, 2017), the study's methodological rigor and varied contexts strengthen the applicability of its findings.

### Implications for Practice

The study found low awareness of CC in both urban and rural areas, underscoring the need for targeted interventions. These results provide baseline data to guide tailored health programs and inform policies that promote screening and vaccination among both adults and children. Such initiatives can increase preventive service use, helping Tanzania and other LMICs in SSA work toward the WHO elimination target and reduce CC-related morbidity and mortality (WHO, 2022).

## Conclusions

The study revealed that both men and women in urban and rural Tanzania had limited knowledge and several misconceptions about CC, its causes, screening, care, and HPV vaccination. Awareness of HPV and its preventive role was low, and beliefs like the vaccine causing infertility or CC being hereditary and untreatable were common. Sociocultural factors as gender norms, fear of abandonment, and lack of parental involvement, strongly influenced awareness and attitudes. Structural barriers, including long distances to health facilities, financial constraints, and limited outreach, further hindered access. Male partners and community opinion leaders were identified as key influencers, yet they lacked adequate information. The findings highlight an issue of low health literacy and the urgent need for community-based education and improved health communication to support participation in CC prevention.

To address these gaps, integrating CC education into school curricula for both girls and boys would promote early awareness, support HPV vaccination uptake, and reduce future disease burden in line with WHO's 90-70-90 targets and Sustainable Development Goal (SDG) 3 (United Nations, 2015). Healthcare providers should counter prevailing myths with evidence-based information, while strengthening women's health literacy supports gender equality and informed decision-making (SDG 5). Training trusted community stakeholders, local leaders, religious figures, and community health workers in culturally sensitive CC prevention can improve community trust and uptake of screening and vaccination, contributing to national and global CC elimination goals. Overall, this study adds important evidence on community perspectives and barriers to CC prevention in both rural and urban contexts in Tanzania, underscoring the need for tailored interventions that can strengthen awareness, promote equitable access, and ultimately reduce the CC burden in the country and comparable settings.

## Appendix 1. COnsolidated criteria for REporting Qualitative research (COREQ) Checklist

A checklist of items that should be included in reports of qualitative research. You must report the page number in your manuscript where you consider each of the items listed in this checklist. If you have not included this information, either revise your manuscript accordingly before submitting or note N/A.

**Table:**

Topic	Item No.	Guide questions/description	Reported on page No.
Domain 1: Research team and reflexivity			
Personal characteristics			
Interviewer/facilitator	1	Which author/s conducted the interview or focus group?	
Credentials	2	What were the researcher's credentials? For example, PhD, MD	
Occupation	3	What was their occupation at the time of the study?	
Gender	4	Was the researcher male or female?	
Experience and training	5	What experience or training did the researcher have?	
Relationship with participants			
Relationship established	6	Was a relationship established prior to study commencement?	
Participant knowledge of the interviewer	7	What did the participants know about the researcher? For example, personal goals, reasons for doing the research	
Interviewer characteristics	8	What characteristics were reported about the inter viewer/facilitator? For example Bias, assumptions, reasons and interests in the research topic	
Domain 2: Study design			
Theoretical framework			
Methodological orientation and theory	9	What methodological orientation was stated to underpin the study? For example, grounded theory, discourse analysis, ethnography, phenomenology, content analysis	
Participant selection			
Sampling	10	How were participants selected? For example, purposive, convenience, consecutive, snowball	
Method of approach	11	How were participants approached? For example, face-to-face, telephone, mail, email	
Sample size	12	How many participants were in the study?	
Nonparticipation	13	How many people refused to participate or dropped out? Reasons?	
Setting			
Setting of data collection	14	Where was the data collected? For example, home, clinic, workplace	
Presence of nonparticipants	15	Was anyone else present besides the participants and researchers?	
Description of sample	16	What are the important characteristics of the sample? For example, demographic data, date	
Data collection			
Interview guide	17	Were questions, prompts, guides provided by the authors? Was it pilot tested?	
Repeat interviews	18	Were repeat inter views carried out? If yes, how many?	
Audio/visual recording	19	Did the research use audio or visual recording to collect the data?	
Field notes	20	Were field notes made during and/or after the interview or focus group?	
Duration	21	What was the duration of the inter views or focus group?	
Data saturation	22	Was data saturation discussed?	
Transcripts returned	23	Were transcripts returned to participants for comment and/or	
Topic	Item No.	Guide questions/description	Reported on page No.
		correction?	
Domain 3: analysis and findings			
Data analysis			
Number of data coders	24	How many data coders coded the data?	
Description of the coding tree	25	Did authors provide a description of the coding tree?	
Derivation of themes	26	Were themes identified in advance or derived from the data?	
Software	27	What software, if applicable, was used to manage the data?	
Participant checking	28	Did participants provide feedback on the findings?	
Reporting			
Quotations presented	29	Were participant quotations presented to illustrate the themes/findings? Was each quotation identified? For example, participant number	
Data and findings consistent	30	Was there consistency between the data presented and the findings?	
Clarity of major themes	31	Were major themes clearly presented in the findings?	
Clarity of minor themes	32	Is there a description of diverse cases or discussion of minor themes?	

*Source.* Developed from: Tong A, Sainsbury P, Craig J. Consolidated criteria for reporting qualitative research (COREQ): A 32-item checklist for interviews and focus groups. *International Journal for Quality in Health Care*. 2007. Volume 19, Number 6: pp. 349–357.

Once you have completed this checklist, please save a copy and upload it as part of your submission. DO NOT include this checklist as part of the main manuscript document. It must be uploaded as a separate file.

## Appendix 2.

Interview Guide FGD

"Cervical Cancer—A Silent Disease in the Community”—A Qualitative Study on Awareness of Cervical Cancer in Tanzania

Introduction and warm-up:

Thank you for coming to the Focus Group today. First, we will collect sociodemographic details by filling in the piece of paper provided. You will identify with yourselves using the numbers provided to each of you, visible to everyone. You are encouraged to speak freely and share your personal opinions and experiences from your community. Remember, there is no right or wrong response.

### A. Sociodemographic Characteristics

Gender……, Age……, Education………, occupation ……, Parity…… Zone………,

Urban /Rural ………

### B. Awareness in Seeking Cervical Cancer Prevention

1. What do you know about cervical cancer (CC) prevention?
2. For your opinion, do you think are there any benefit for CC prevention?

If yes, what are the benefits of CC prevention? If no, why?

3. Are you aware about any preventive measures for CC prevention?

**Probe:** HPV vaccination, screening for CC, if not mentioned

Awareness about signs and symptoms, what causes CC? Who is at risk of being infected with CC?

4. Are you aware that CC can be prevented? If yes, how can be prevented? If no, do you think why cannot be prevented? **Probe:** Primary prevention; secondary prevention
5. Who is eligible to be screened for CC? Why?
6. Are you aware on the vaccination recommended for CC?

**Probe:** Who to be vaccinated, at what age, doses for each eligible for vaccination

7. Are you aware about the availability of screening services and treatment of precancer services?

Attitudes toward HPV-vaccination for young girls

8. Have you ever thought on vaccinating young girls against HPV to prevent CC? Why?

**Probe more on**: + expectation for vaccine, and + attitudes.

### C. Barriers and Facilitators in Seeking Prevention Measures

Barriers for seeking CA prevention

9. What do you think prohibit most of the women in seeking CC prevention?

**Probe:** On vaccination for young girls (Family, cultural or community barriers, accessibility

**Probe:** Barriers for screening beliefs and attitudes or misconception, health facility barriers, cost of screening (transport and other cost due to distance), emotional cost.

10. How does the community around influence on prevention of CC?

**Probe:** Influence on vaccination acceptance, screening for CC attitudes and beliefs in community

Facilitators in seeking CC prevention

11. What do you think facilitates readiness for attendance for screening among women?

**Probe:** Close family members, husbands, relatives, community, health care provider's initiations, poor health conditions, accessibility of the screening centers, vaccination services, awareness campaign at the community, having health insurance, income.

Closing questions

- Is there anything else you would like to share about cc prevention in your community?
- Do you have any suggestions or questions?

Thank you very much for your time participating in this FGD.

End of the discussion

## References

[bibr1-23779608251393079] Adedimeji A. Ajeh R. Pierz A. Nkeng R. Ndenkeh J. J. Fuhngwa N. Nsame D. Nji M. Dzudie A. Anastos K. M. Castle P. E. (2021). Challenges and opportunities associated with cervical cancer screening programs in a low-income, high HIV prevalence context. BMC Women's Health, 21(18), 1–14. 10.1186/s12905-021-01211-w 33602194 PMC7890622

[bibr2-23779608251393079] Adewumi K. Oketch S. Y. Choi Y. Huchko M. J. (2019). Female perspectives on male involvement in a human-papillomavirus-based cervical cancer-screening program in western Kenya. BMC Women's Health, 19(1), 1–9. 10.1186/s12905-019-0804-4 31395060 PMC6688365

[bibr3-23779608251393079] Adler A. J. Randall T. Schwartz L. N. Drown L. Matthews S. Pace L. E. Mugabo C. Kateera F. Bukhman G. Baganizi E. Ng'ang'a L. M. (2023). What women want: A mixed-methods study of women's health priorities, preferences, and experiences in care in three Rwandan rural districts. International Journal of Gynecology & Obstetrics, 162(2), 525–531. 10.1002/ijgo.14735 36815725

[bibr4-23779608251393079] Agimas M. C. Adugna D. G. Derseh N. M. Kassaw A. Kassie Y. T. Abate H. K. Mekonnen C. K. (2024). Uptake of human papillomavirus vaccine and its determinants among females in east Africa: A systematic review and meta-analysis. BMC Public Health, 24(1), Article 842. 10.1186/s12889-024-18141-5 38500046 PMC10949808

[bibr5-23779608251393079] Almehmadi M. M. Salih M. M. Al-Hazmi A. S. (2019). Awareness of human papillomavirus infection complications, cervical cancer, and vaccine among the Saudi population: A cross-sectional survey. Saudi Medical Journal, 40(6), Article 555. 10.15537/smj.2019.6.24208 31219489 PMC6778758

[bibr6-23779608251393079] Anyaka C. U. Alero B. J. Olukoya B. Envuladu E. A. Musa J. Sagay A. S. (2024). Parental knowledge of HPV infection, cervical cancer, and the acceptance of HPV vaccination for their children in Jos, Nigeria. Journal of West African College of Surgeons, 14(2), 146–153. 10.4103/jwas.jwas_309_22 PMC10980327 38562384

[bibr7-23779608251393079] Appiah R. S. Boakye K. Appiah G. Aidoo A. A. Acquah-Hagan G. Singh B. Appiah F. Boateng D. (2025). Rural-urban variations in cervical cancer screening uptake among women in Ghana: Evidence from the 2022 Ghana Demographic and Health Survey. BMC Women's Health, 25(1), Article 255. 10.1186/s12905-025-03802-3 40420073 PMC12105228

[bibr8-23779608251393079] Arbyn M. Weiderpass E. Bruni L. de Sanjosé S. Saraiya M. Ferlay J. Bray F. (2020). Estimates of incidence and mortality of cervical cancer in 2018: A worldwide analysis. The Lancet Global Health, 8(2), e191–e203. 10.1016/S2214-109X(19)30482-6 PMC7025157 31812369

[bibr9-23779608251393079] Asgedom Y. S. Hailegebireal A. H. Woldegeorgis B. Z. Koyira M. M. Seifu B. L. Fente B. M. Gebrekidan A. Y. Tekle H. A. Asnake A. A. Kassie G. A. (2024). Towards 90-70-90 targets: Individual and community level factors associated with cervical cancer screening among women of reproductive age in Tanzania: A multi-level analysis based on the 2022 Tanzania demographic and health survey. Plos one, 19(12), e0315438. 10.1371/journal.pone.0315438 PMC11654981 39693312

[bibr10-23779608251393079] Baharum N. N. Ariffin F. Isa M. R. Tin S. T. (2020). Health literacy, knowledge on cervical cancer and pap smear and its influence on pre-marital Malay Muslim women attitude towards pap smear. Asian Pacific Journal of Cancer Prevention: APJCP, 21(7), Article 2021. 10.31557/APJCP.2020.21.7.2021 32711428 PMC7573396

[bibr11-23779608251393079] Banks K. S. James C. M. Nganwa D. Heath J. Webb L. Elhussin I. Faraj R. Abdalla E. (2022). Knowledge and awareness about cervical cancer and human papillomavirus among women living in Macon county, Alabama. Journal of Healthcare, Science and the Humanities, 12(1), 13. PMID: 37465463. 37465463 PMC10351483

[bibr12-23779608251393079] Barrow A. Onikan A. Nzoputam C. I. Ekholuenetale M. (2020). Prevalence and determinants of cervical cancer awareness among women of reproductive age: Evidence from Benin and Zimbabwe population-based data. Applied Cancer Research, 40(1), 1–13. 10.1186/s41241-020-00092-z

[bibr13-23779608251393079] Bateman L. B. Blakemore S. Koneru A. Mtesigwa T. McCree R. Lisovicz N. F. Aris E. A. Yuma S. Mwaiselage J. D. Jolly P. E. (2019). Barriers and facilitators to cervical cancer screening, diagnosis, follow-up care and treatment: Perspectives of human immunodeficiency virus-positive women and health care practitioners in Tanzania. The Oncologist, 24(1), 69–75. 10.1634/theoncologist.2017-0444 29934410 PMC6324638

[bibr14-23779608251393079] Behr D. (2017). Assessing the use of back translation: The shortcomings of back translation as a quality testing method. International Journal of Social Research Methodology, 20(6), 573–584. 10.1080/13645579.2016.1252188

[bibr15-23779608251393079] Bénard É Drolet M. Laprise J. F. Gingras G. Jit M. Boily M. C. Bloem P. Brisson M. (2023). Potential population-level effectiveness of one-dose HPV vaccination in low-income and middle-income countries: A mathematical modelling analysis. The Lancet Public Health, 8(10), e788–e799. 10.1016/S2468-2667(23)00180-9 PMC10557953 37777288

[bibr16-23779608251393079] Bensemmane S. Loayza Villarroel K. Montaño K. Louati E. Ascarrunz C. Rodriguez P. Fontaine V. Laokri S. (2022). Assessing barriers encountered by women in cervical cancer screening and follow-up care in urban Bolivia, Cochabamba. In Healthcare (Vol. 10, No. 9, p. 1604). MDPI. 10.3390/healthcare10091604 PMC9498362 36141216

[bibr17-23779608251393079] Binka C. Doku D. T. Awusabo-Asare K. (2017). Experiences of cervical cancer patients in rural Ghana: An exploratory study. PloS one, 12(10), Article e0185829. 10.1371/journal.pone.0185829 PMC5636100 29020099

[bibr18-23779608251393079] Bray F. Ferlay J. Soerjomataram I. Siegel R. L. Torre L. A. Jemal A. (2018). Global cancer statistics 2018: GLOBOCAN estimates of incidence and mortality worldwide for 36 cancers in 185 countries. CA: A Cancer Journal for Clinicians, 68(6), 394–424. 10.3322/caac.21492 30207593

[bibr19-23779608251393079] Bruni L. Albero G. Serrano B. Mena M. Collado J. J. Gómez D. Muñoz J. Bosch F. X. de Sanjosé S. (2019). Information centre on HPV and cancer (HPV Information Centre). Human Papillomavirus and related diseases in the world. Summary Report 10 March 2023.

[bibr20-23779608251393079] Bruni L. Serrano B. Roura E. Alemany L. Cowan M. Herrero R. Poljak M. Murillo R. Broutet N. Riley L. M. de Sanjose S. (2022). Cervical cancer screening programmes and age-specific coverage estimates for 202 countries and territories worldwide: A review and synthetic analysis. The Lancet Global Health, 10(8), e1115–e1127. 10.1016/S2214-109X(22)00241-8 PMC9296658 35839811

[bibr21-23779608251393079] Canfell K. Kim J. J. Brisson M. Keane A. Simms K. T. Caruana M. Burger E. A. Martin D. Nguyen D. T. Bénard É Sy S. (2020). Mortality impact of achieving WHO cervical cancer elimination targets: Comparative modeling analysis in 78 low-income and lower-middle-income countries. The Lancet, 395(10224), 591–603. 10.1016/S0140-6736(20)30157-4 PMC7043006 32007142

[bibr22-23779608251393079] Centre for Disease Control and Prevention, About Rural health . (2024). https://www.cdc.gov/rural-health/php/about/index.html .

[bibr23-23779608251393079] Chapola J. Lee F. Bula A. Mapanje C. Phiri B. R. Kamtuwange N. Tsidya M. Tang J. H. Chinula L. (2021). Barriers to follow-up after an abnormal cervical cancer screening result and the role of male partners: A qualitative study. BMJ open, 11(9), Article e049901. 10.1136/bmjopen-2021-049901 PMC8442050 34521669

[bibr24-23779608251393079] Chelva M. Kaushal S. West N. Erwin E. Yuma S. Sleeth J. Yahya-Malima K. I. Shelley D. Risso-Gill I. Yeates K. (2024). “In the village that she comes from, most of the people don’t know anything about cervical cancer”: A health systems appraisal of cervical cancer prevention services in Tanzania. International Journal of Environmental Research and Public Health, 21(8), Article 1059. 10.3390/ijerph21081059 39200668 PMC11353714

[bibr25-23779608251393079] Chona E. Z. Msengi E. A. Gosse R. A. Ambikile J. S. (2023). The lived experiences and caring needs of women diagnosed with cervical cancer: A qualitative study in Dar es Salaam, Tanzania. PloS one, 18(8), Article e0289925. 10.1371/journal.pone.028992 PMC10414621 37561728

[bibr26-23779608251393079] Cinya R. Mubangizi P. (2024). A cross-sectional study on factors contributing to low uptake of human papillomavirus vaccination among females aged 10-12 years attending Adjumani General Hospital, Adjumani district. SJ Public Health Africa, 1(4), 15–15. 10.51168/x9kzhk51

[bibr27-23779608251393079] Cooper E. C. Maher J. A. Naaseh A. Crawford E. W. Chinn J. O. Runge A. S. Lucas A. N. Zezoff D. C. Bera K. R. Dinicu A. I. White K. M. Tewari S. E. Hari A. Bernstein M. Chang J. Ziogas A. Pearre D. C. Tewari K. S. (2021). Implementation of human papillomavirus video education for women participating in mass cervical cancer screening in Tanzania. American Journal of Obstetrics and Gynecology, 224(1), 105–1e1. 10.1016/j.ajog.2020.07.018 32682861

[bibr28-23779608251393079] Creswell J. W. Poth C. N. (2016). Qualitative inquiry and research design: Choosing among five approaches. Sage Publications.

[bibr29-23779608251393079] Cunningham M. S. Skrastins E. Fitzpatrick R. Jindal P. Oneko O. Yeates K. Booth C. M. Carpenter J. Aronson K. J. (2015). Cervical cancer screening and HPV vaccine acceptability among rural and urban women in Kilimanjaro region, Tanzania. BMJ open, 5(3), Article e005828. 10.1136/bmjopen-2014-005828 PMC4360576 25757944

[bibr30-23779608251393079] Davies C. Stoney T. Hutton H. Parrella A. Kang M. Macartney K. Leask J. McCaffery K. Zimet G. Brotherton J. M. L. Marshall H. S. Skinner S. R. (2021). School-based HPV vaccination positively impacts parents’ attitudes toward adolescent vaccination. Vaccine, 39(30), 4190–4198. 10.1016/j.vaccine.2021.05.051 34127299

[bibr31-23779608251393079] Defo V. F. Domgue J. F. (2020). Why consider self-sampling for cervical cancer screening in low- and middle-income countries? AMA journal of Ethics, 22(2), 116–125. 10.1001/amajethics.2020.116 32048582

[bibr32-23779608251393079] de Fouw M. Stroeken Y. Niwagaba B. Musheshe M. Tusiime J. Sadayo I. Reis R. Peters A. A. W. Beltman J. J. (2023). Involving men in cervical cancer prevention; a qualitative enquiry into male perspectives on screening and HPV vaccination in mid-western Uganda. PLoS One, 18(1), Article e0280052. 10.1371/journal.pone.0280052 PMC9882699 36706114

[bibr33-23779608251393079] Demissie B. W. Azeze G. A. Asseffa N. A. Lake E. A. Besha B. B. Gelaw K. A. Mokonnon T. M. Gebeyehu N. A. Obsa M. S. (2022). Communities’ perceptions towards cervical cancer and its screening in Wolaita zone, southern Ethiopia: A qualitative study. PloS one, 17(1), e0262142. 10.1371/journal.pone.0262142 PMC8740975 34995307

[bibr34-23779608251393079] Dirar A. Mekonnen W. Berhanu Z. (2022). The experiences of cervical cancer patients during follow-up care in Ethiopia: A qualitative study. Cancer Management and Research, 2022(14), 2507–2518. 10.2147/CMAR.S373379 PMC9416456 36035503

[bibr35-23779608251393079] Drokow E. K. Zi L. Han Q. Effah C. Y. Agboyibor C. Sasu E. Akpabla G. S. Foli F. Sun K. (2020). Awareness of cervical cancer and attitude toward human papillomavirus and its vaccine among Ghanaians. Frontiers in Oncology, 10, Article 1651. 10.3389/fonc.2020.01651 33014828 PMC7506130

[bibr36-23779608251393079] Duncan K. (2022). Facilitators enabling sustainable cervical cancer control programs in low-and middle-income countries: Strengthening health systems in Zambia [Doctoral dissertation]. The University of North Carolina at Chapel Hill.

[bibr37-23779608251393079] Elo S. Kääriäinen M. Kanste O. Pölkki T. Utriainen K. Kyngäs H. (2014). Qualitative content analysis: A focus on trustworthiness. SAGE Open, 4(1), 1–10. 10.1177/2158244014522633

[bibr38-23779608251393079] Gavi the Vaccine Alliance. (2021). Human papillomavirus vaccine support. Available at: https://www.gavi.org/types-support/vaccine-support/human-papillomavirus (Accessed: 14 August 2025).

[bibr39-23779608251393079] Gesink M. P. Chamberlain R. M. Mwaiselage J. Kahesa C. Jackson K. Mueller W. Meza J. L. Soliman A. S. (2020). Quantifying the underestimation of cervical cancer in remote regions of Tanzania. BMC cancer, 20(1), 1–8. 10.1186/s12885-020-07439-3 PMC7526175 32998702

[bibr40-23779608251393079] Gizaw A. T. El-Khatib Z. Wolancho W. Amdissa D. Bamboro S. Boltena M. T. Appiah S. C. Y. Asamoah B. O. Wasihun Y. Tareke K. G. (2022). Uptake of cervical cancer screening and its predictors among women of reproductive age in Gomma district, southwest Ethiopia: A community-based cross-sectional study. Infectious Agents and Cancer, 17(1), Article 43. 10.1186/s13027-022-00455-x 35941664 PMC9358816

[bibr41-23779608251393079] Graneheim U. H. Lundman B. (2004). Qualitative content analysis in nursing research: Concepts, procedures, and measures to achieve trustworthiness. Nurse Education Today, 24(2), 105–112. 10.1016/j.nedt.2003.10.001 14769454

[bibr42-23779608251393079] Griesel M. Seraphin T. P. Mezger N. C. Hämmerl L. Feuchtner J. Joko-Fru W. Y. Sengayi-Muchengeti M. Liu B. Vuma S. Korir A. Chesumbai G. C. (2021). Cervical cancer in Sub-Saharan Africa: A multinational population-based cohort study of care and guideline adherence. The Oncologist, 26(5), e807–e816. 10.1002/onco.13718 PMC8100544 33565668

[bibr43-23779608251393079] Gultekin M. Ramirez P. T. Broutet N. Hutubessy R. (2020). World health organization calls for action to eliminate cervical cancer globally. International Journal of Gynecological Cancer, 30(4), 426–427. 10.1136/ijgc-2020-001285 32122950

[bibr44-23779608251393079] Hasahya O. T. Berggren V. Sematimba D. Nabirye R. C. Kumakech E. (2016). Beliefs, perceptions and health-seeking behaviours in relation to cervical cancer: A qualitative study among women in Uganda following completion of an HPV vaccination campaign. Global Health Action, 9(1), Article 29336. 10.3402/gha.v9.29336 26895145 PMC4759844

[bibr45-23779608251393079] Ho Y. C. L. Mahirah D. Ho C. Z. H. Thumboo J. (2022). The role of the family in health promotion: A scoping review of models and mechanisms. Health Promotion International, 37(6), Article daac119. 10.1093/heapro/daac119 PMC9673498 36398941

[bibr46-23779608251393079] Hussein K. Kokwaro G. Wafula F. Kassie G. M. (2024). Assessing the influence of the health system on access to cervical cancer prevention, screening, and treatment services at public health centers in Addis Ababa, Ethiopia. Plos one, 19(5), Article e0300152. 10.1371/journal.pone.0300152 PMC11142424 38820249

[bibr47-23779608251393079] Isaacson S. Adewumi K. Smith J. S. Novak C. Oketch S. Huchko M. J. (2023). A qualitative exploration of barriers to treatment among HPV-positive women in a cervical cancer screening study in western Kenya. The Oncologist, 28(1), e9–e18. 10.1093/oncolo/oyac208 PMC9847557 36239434

[bibr48-23779608251393079] Jibat N. Ali R. Adissu W. Buruh G. Abdissa A. Goba G. K. Garland S. M. Mulholland N. Mulholland K. Amenu D. (2024). Less known but greatly feared: Cervical cancer in Ethiopia, community awareness. Heliyon, 10(7), 1–10. 10.1016/j.heliyon.2024.e28328 PMC11004701 38601557

[bibr49-23779608251393079] Kilimanjaro Christian Medical Centre, Cancer Care Centre . (2022.

[bibr50-23779608251393079] Krueger R. A. Casey M. A. (2015). Focus group interviewing. Handbook of Practical Program Evaluation, 506–534. 10.1002/9781119171386.ch20

[bibr51-23779608251393079] Lin W. Wang Y. Liu Z. Chen B. Yuan S. Wu B. Gong L. (2020). Inequalities in awareness and attitude towards HPV and its vaccine between local and migrant residents who participated in cervical cancer screening in Shenzhen, China. Cancer Research and Treatment: Official Journal of Korean Cancer Association, 52(1), 207–217. 10.4143/crt.2019.053 PMC6962482 31291712

[bibr52-23779608251393079] Lincoln Y. S. Guba E. G. (1985). Naturalistic inquiry. Sage Publications.

[bibr53-23779608251393079] Mantula F. Toefy Y. (2023). Women and health providers’ perspectives on male support for cervical cancer screening in Gwanda district, Zimbabwe. Plos one, 18(10), Article e0282931. 10.1371/journal.pone.0282931 PMC10569579 37824479

[bibr54-23779608251393079] Marshall C. Rossman G. B. (2016). Designing qualitative research (6th ed.). Sage Publications.

[bibr55-23779608251393079] Mihretie G. N. Liyeh T. M. Ayele A. D. Belay H. G. Yimer T. S. Miskr A. D. (2022). Knowledge and willingness of parents towards child girl HPV vaccination in Debre Tabor town, Ethiopia: A community-based cross-sectional study. Reproductive Health, 19(1), Article 136. 10.1186/s12978-022-01444-4 35689288 PMC9188100

[bibr56-23779608251393079] Morse R. M. Brown J. Gage J. C. Prieto B. A. Jurczuk M. Matos Vásquez Vásquez J. Reátegui R. R. Meza-Sanchez G. Córdova L. A. Gravitt P. E. A. Paz-Soldan V. A. (2023). “Easy women get it”: Pre-existing stigma associated with HPV and cervical cancer in a low-resource setting prior to implementation of an HPV screen-and-treat program. BMC Public Health, 23(1), Article 2396. 10.1186/s12889-023-17324-w 38042779 PMC10693157

[bibr57-23779608251393079] Moshi F. V. Vandervort E. B. Kibusi S. M. (2018). Cervical cancer awareness among women in Tanzania: An analysis of data from the 2011-12 Tanzania HIV and Malaria Indicators Survey. International journal of chronic diseases, 2018(2458232), 1–7. 10.1155/2018/2458232 PMC5954879 29854721

[bibr58-23779608251393079] Mphuru A. Li A. J. Kyesi F. Mwengee W. Mazige F. Nshunju R. Shayo B. Giattas M. R. Loharikar A. Lyimo D. (2022). National introduction of human papillomavirus (HPV) vaccine in Tanzania: Programmatic decision-making and implementation. Vaccine, 40(Suppl 1), A2–A9. 10.1016/j.vaccine.2021.04.025 PMC9601814 33962839

[bibr59-23779608251393079] Nhumba N. W. Sunguya B. (2022). Low uptake of the second dose of human papillomavirus vaccine in Dar es Salaam, Tanzania. Vaccines, 10(11), Article 1919. 10.3390/vaccines10111919 36423015 PMC9695747

[bibr60-23779608251393079] Osei E. A. Appiah S. Gaogli J. E. Oti-Boadi E. (2021). Knowledge on cervical cancer screening and vaccination among females at oyibi community. BMC Women's Health, 22(1), 1–9. 10.1186/s12905-021-01296-3 PMC8042702 33845829

[bibr61-23779608251393079] Patel H. Sherman S. M. Tincello D. Moss E. L. (2020). Awareness of and attitudes towards cervical cancer prevention among migrant eastern European women in England. Journal of Medical Screening, 27(1), 40–47. 10.1177/0969141319869957 31514572

[bibr62-23779608251393079] Petersen Z. Jaca A. Ginindza T. G. Maseko G. Takatshana S. Ndlovu P. Zondi N. Zungu N. Varghese C. Hunting G. Parham G. (2022). Barriers to uptake of cervical cancer screening services in low-and-middle-income countries: A systematic review. BMC women's Health, 22(1), Article 486. 10.1186/s12905-022-02043-y 36461001 PMC9716693

[bibr63-23779608251393079] Polit D. F. Beck C. T. (2017). Key concepts and steps in qualitative and quantitative research. Nursing Research. Generating and Assessing Evidence for Nursing Practice, DF Polit and CT Beck, Editors.

[bibr64-23779608251393079] Ragan K. R. Buchanan Lunsford N. Lee Smith J. Saraiya M. Aketch M. (2018). Perspectives of screening-eligible women and male partners on benefits of and barriers to treatment for precancerous lesions and cervical cancer in Kenya. The Oncologist, 23(1), 35–43. 10.1634/theoncologist.2017-0053 28798272 PMC5759810

[bibr65-23779608251393079] Rick T. J. Deming C. M. Helland J. R. Hartwig K. A. (2019). Cancer training for frontline healthcare providers in Tanzania. Journal of Cancer Education, 34(1), 111–115. 10.1007/s13187-017-1274-8 28815475

[bibr66-23779608251393079] Runge A. S. Bernstein M. E. Lucas A. N. Tewari K. S. (2019). Cervical cancer in Tanzania: A systematic review of current challenges in six domains. Gynecologic Oncology Reports, 29, 40–47. 10.1016/j.gore.2019.05.008 31309135 PMC6606891

[bibr67-23779608251393079] Shrestha G. Mulmi R. Phuyal P. Thakur R. K. Siwakoti B. (2020). Experiences of cervical cancer survivors in Chitwan, Nepal: A qualitative study. PloS one, 15(11), 1–14. 10.1371/journal.pone.0234834N PMC7644025 33151965

[bibr68-23779608251393079] Simelela P. N. (2021). WHO global strategy to eliminate cervical cancer as a public health problem: An opportunity to make it a disease of the past. Int. J Gynecol Obstet, 152(1), 1–3. 10.1002/ijgo.13484 33269466

[bibr69-23779608251393079] Srinath A. van Merode F. Rao S. V. Pavlova M. (2023). Barriers to cervical cancer and breast cancer screening uptake in low-and middle-income countries: A systematic review. Health Policy and Planning, 38(4), 509–527. 10.1093/heapol/czac104 36525529 PMC10089064

[bibr70-23779608251393079] Stefan D. C. Dangou J. M. Barango P. Mahamadou I. D. Kapambwe S. (2022). The World Health Organization targets for cervical cancer control by 2030: A baseline assessment in six African countries—part I. Ecancermedicalscience, 16, 1453. 10.3332/ecancer.2022.1453 36405945 PMC9666282

[bibr71-23779608251393079] Stenfors T. Kajamaa A. Bennett D. (2020). How to … assess the quality of qualitative research. The Clinical Teacher, 17(6), 596–599. 10.1111/tct.13242 32790137

[bibr72-23779608251393079] Swai P. Mgongo M. Leyaro B. J. Mwaiselage J. Mchome B. L. Kjaer S. K. Rasch V. Manongi R. Msuya S. E. (2023). Knowledge on human papilloma virus and experience of getting positive results: A qualitative study among women in Kilimanjaro, Tanzania. BMC Women's Health, 23(1), Article 61. 10.1186/s12905-023-02192-8 36774477 PMC9921604

[bibr73-23779608251393079] Tapera O. Dreyer G. Kadzatsa W. Nyakabau A. M. Stray-Pedersen B. Sjh H. (2019). Cervical cancer knowledge, attitudes, beliefs and practices of women aged at least 25 years in Harare, Zimbabwe. BMC Women's Health, 19, 1–10. 10.1186/s12905-019-0790-6 31286937 PMC6615311

[bibr74-23779608251393079] Tapera O. Dreyer G. Nyakabau A. M. Kadzatsa W. Stray-Pedersen B. Hendricks S. J. H. (2021). Model strategies to address barriers to cervical cancer treatment and palliative care among women in Zimbabwe: A public health approach. BMC women's Health, 21(1), 180. 10.1186/s12905-021-01322-4 33906670 PMC8077905

[bibr75-23779608251393079] Tobaiqy M. MacLure K. (2024). A systematic review of human Papillomavirus vaccination challenges and strategies to enhance uptake. Vaccines, 12(7), 746. 10.3390/vaccines12070746 39066384 PMC11281456

[bibr76-23779608251393079] Tobaiqy M. A. Mehdar S. A. Altayeb T. I. Saad T. M. Alqutub S. T. (2023). Parental knowledge, views, and perceptions of human papilloma virus infection and vaccination-cross-sectional descriptive study. Journal of Family Medicine and Primary Care, 12(3), 556–560. 10.4103/jfmpc.jfmpc_1673_22 PMC10131950 37122659

[bibr77-23779608251393079] Tong A. Sainsbury P. Craig J. (2007). Consolidated criteria for reporting qualitative research (COREQ): A 32-item checklist for interviews and focus groups. International Journal for Quality in Health Care, 19(6), 349–357. 10.1093/intqhc/mzm042 17872937

[bibr78-23779608251393079] United Nations (2015). Transforming our world: The 2030 agenda for sustainable development. Available at: https://sdgs.un.org/2030agenda (Accessed: 14 August 2025).

[bibr79-23779608251393079] United Republic of Tanzania Ministry of Health and Social Welfare . (2015). National Cervical Cancer Prevention and Control Strategic Plan (NCCPCSP) 2011–2015. Available at: https://extranet.who.int/ncdccs/Data/TZA_B5_Cervical%20Cancer%20Strategic%20Plan2011.pdf.

[bibr80-23779608251393079] World Health Organization . (2020). Global strategy to accelerate the elimination of cervical cancer as a public health problem. Geneva: World Health Organization. Available at: https://www.who.int/publications/i/item/9789240014107 (Accessed: 14 August 2025).

[bibr81-23779608251393079] World Health Organization . (2022). Accelerating the elimination of cervical cancer as a public health problem: Towards achieving 90–70–90 targets by 2030. In accelerating the elimination of cervical cancer as a public health problem: Towards achieving 90–70–90 targets by 2030.

[bibr82-23779608251393079] World Health Organization . (2024). Considerations for human papillomavirus (HPV) vaccine product choice. World Health Organization.2024 Apr 17. https://www.who.int https://www.who.int/groups/global-advisory-committee-on-vaccine-safety/topics/human-papillomavirus-vaccines/safety-of-hpv.

[bibr83-23779608251393079] World Medical Association . (2013). World Medical Association declaration of Helsinki: Ethical principles for medical research involving human subjects. JAMA, 310(20), 2191–2194. 10.1001/jama.2013.281053 24141714

[bibr84-23779608251393079] Zhetpisbayeva I. Kassymbekova F. Sarmuldayeva S. Semenova Y. Glushkova N. (2023). Cervical cancer prevention in rural areas. Annals of Global Health, 89(1), Article 75. 10.5334/aogh.4133 37928103 PMC10624144

